# Mendelian randomization reveals causal circulatory microRNAs in prostate cancer pathogenesis and prognosis

**DOI:** 10.1097/MD.0000000000043613

**Published:** 2025-08-01

**Authors:** Zhicheng Cong, Dandan Qiu, Haiping Hu

**Affiliations:** a Department of Urology, Zhejiang Hospital, Hangzhou, Zhejiang, China; b Department of Urology, The First Affiliated Hospital of Zhejiang Chinese Medical University, Hangzhou, Zhejiang, China.

**Keywords:** expression quantitative trait loci, Mendelian randomization, miRNA, prostate cancer, survival

## Abstract

Prostate cancer (PCa) lacks precise diagnostic tools, necessitating noninvasive biomarkers. MicroRNAs (miRNAs) show promise due to fluid stability and oncogenic regulation. Using 2-sample Mendelian randomization (MR), we analyzed plasma miRNA expression quantitative trait loci (eQTLs) from discovery and validation cohorts. Cis-acting eQTLs served as instrumental variables, with causal effects assessed via inverse-variance weighted (IVW) and MR-Egger regression. Functional and survival analyses leveraged The Cancer Genome Atlas (TCGA)-PRAD data and bioinformatics tools. Five miRNAs (hsa-miR-127-3p, -370-3p, -125a-5p, -433-3p, -99b-5p) were inversely associated with PCa risk (IVW *P* < .05, FDR < 0.1). Two (hsa-miR-125a-5p/-99b-5p) were validated (OR = 0.992–0.9924, *P* < .05). Their 553 target genes included 9 survival-linked genes (e.g., FOXM1, BTG2), enriched in basement membrane organization, glutathione metabolism, and senescence. hsa-miR-127-3p was downregulated in PCa (fold change = 0.7, *P* < .001), with pan-cancer prognostic relevance. hsa-miR-125a-5p and -99b-5p are causal protective factors against PCa, modulating oncogenic pathways and survival. These miRNAs offer potential as noninvasive biomarkers and therapeutic targets, advancing precision oncology.

## 1. Introduction

Prostate cancer (PCa) remains one of the most prevalent malignancies among men worldwide, with over 1.4 million new cases diagnosed annually and approximately 375,000 deaths reported in 2020 alone.^[1]^ Despite advances in screening and diagnostic tools, such as prostate-specific antigen (PSA) testing and digital rectal examination (DRE), these methods lack sufficient sensitivity and specificity, often leading to overdiagnosis and overtreatment.^[2]^ Furthermore, current prognostic strategies rely heavily on invasive procedures like biopsies and imaging, which are not only uncomfortable for patients but also fail to provide a comprehensive understanding of tumor heterogeneity and progression.^[3]^ The limitations of existing diagnostic and prognostic tools underscore the urgent need for noninvasive biomarkers that can accurately detect PCa at early stages, predict disease progression, and monitor treatment response. In this context, microRNAs (miRNAs) have emerged as promising candidates due to their stability in bodily fluids, tissue-specific expression, and involvement in key oncogenic pathways.^[4,5]^

miRNAs are small, noncoding RNA molecules that regulate gene expression by binding to target mRNAs, leading to their degradation or translational repression.^[6]^ In cancer, miRNAs can function as either oncogenes (oncomiRs) or tumor suppressors, depending on their target genes and the cellular context.^[7]^ For instance, miR-21 and miR-375 have been consistently reported as upregulated in PCa, promoting tumor growth and metastasis, while miR-34a and miR-145 act as tumor suppressors by inhibiting cell proliferation and inducing apoptosis.^[8,9]^ The dysregulation of miRNAs in PCa is not only associated with tumor initiation and progression but also with treatment resistance, making them valuable biomarkers for disease management.^[10]^ Moreover, miRNAs are secreted into circulation, either bound to proteins or encapsulated in exosomes, allowing their detection in noninvasive liquid biopsies such as plasma, serum, and urine.^[11]^ This characteristic positions miRNAs as ideal candidates for developing novel diagnostic and prognostic tools for PCa.

Despite the potential of miRNAs as biomarkers, several challenges hinder their translation into clinical practice. First, the expression levels of miRNAs in bodily fluids are often low, requiring highly sensitive detection methods such as reverse transcription-quantitative PCR (RT-qPCR) or next-generation sequencing.^[12]^ Second, the lack of standardized protocols for miRNA isolation and quantification has led to variability in results across studies, limiting the reproducibility of findings.^[13]^ Additionally, single miRNAs often exhibit limited specificity for PCa, as their dysregulation is observed in multiple cancer types.^[14]^ To address these limitations, recent studies have focused on developing multi-miRNA panels that combine the predictive power of several miRNAs, often in conjunction with clinical variables like PSA levels.^[15]^ For example, Fredsøe et al developed a urine-based 3-miRNA model that outperformed PSA in predicting biopsy outcomes, highlighting the potential of miRNA-based models for improving PCa diagnosis.^[16]^ These advancements underscore the need for further research to validate miRNA biomarkers and integrate them into clinical workflows.

Mendelian randomization (MR) is a powerful genetic tool that uses genetic variants as instrumental variables (IVs) to infer causal relationships between exposures and outcomes, thereby minimizing confounding and reverse causation.^[17]^ In the context of miRNA research, MR can help identify causal relationships between circulating miRNAs and disease risk, providing insights into the biological mechanisms underlying miRNA dysregulation.^[16,18,19]^ By leveraging large-scale genetic datasets, MR can also address the limitations of observational studies, such as small sample sizes and heterogeneity in miRNA measurement techniques.^[20]^

In this study, we utilized 2 independent plasma circulatory miRNA expression quantitative trait loci (eQTL) datasets to perform MR analysis, aiming to identify potential causal miRNAs for PCa. This approach not only enhances our understanding of miRNA biology in PCa but also paves the way for the development of novel therapeutic targets and diagnostic tools.

## 2. Materials and methods

### 2.1. Data collection and preprocessing

To investigate the potential causal relationships between plasma circulatory miRNAs and PCa, we utilized 2 independent miRNA-eQTL datasets. The discovery cohort was derived from the study by Huan et al,^[21]^ which included 280 high-quality miRNAs and approximately 10 million single nucleotide polymorphisms (SNPs). We focused exclusively on cis-acting miRNA-eQTLs and excluded SNPs located in coding regions with synonymous or missense consequences to minimize potential pleiotropic effects. SNPs with a Benjamini-Hochberg corrected false discovery rate (FDR) < 0.1 were selected as IVs. For validation, we obtained an additional miRNA-eQTL dataset from Nikpay et al,^[22]^ which included miRNA expression profiles from 710 unrelated individuals of European ancestry. To ensure consistency between datasets, miRNA IDs were harmonized using miRCarta v1.1. Furthermore, transcriptomic., mutation, and clinical follow-up data from the TCGA-PRAD cohort, comprising 51 normal and 501 PCa samples, were downloaded from The Cancer Genome Atlas (TCGA, https://portal.gdc.cancer.gov/) database. Finally, PCa genome-wide association study (GWAS) data were retrieved from the IEU Open GWAS Project (https://gwas.mrcieu.ac.uk/, GWAS ID: ukb-b-7773), which included 30,945 cases and 368,725 controls of European ancestry, totaling 9,851,867 SNPs.

### 2.2. MR analysis

Two-sample MR analysis was performed using the R package TwoSampleMR v0.6.8 (14) to assess the genetic associations between miRNAs and PCa. First, linkage disequilibrium analysis was conducted on the IVs in the discovery cohort, with independent IVs identified using an *r*^2^ threshold of < 0.5 and a 10 kb clumping window.^[18]^ SNPs associated with PCa at a significance level of *P* > 5E−08 were excluded. Exposure and outcome data were standardized to ensure alignment of effect alleles, and palindromic SNPs were removed. A miRNA was considered causally associated with PCa if it met the following criteria: a *P*-value < .05 and FDR < 0.1 in the inverse-variance weighted (IVW) test; at least 3 SNPs available for MR analysis; and consistent directionality of effect estimates between MR-Egger and IVW methods. Potential horizontal pleiotropy was assessed using MR-Egger regression, and the robustness of the results was evaluated using leave-one-out analysis to determine the influence of individual SNPs on the overall causal estimate.

### 2.3. miRNA-target analysis and functional enrichment

To explore the biological mechanisms underlying the identified miRNAs, target gene analysis was performed using miRNet 2.0 (https://www.mirnet.ca/miRNet/home.xhtml). Experimentally validated target genes were retrieved from the miRTarBase v9.0 database. The miRNA-gene interaction network was visualized using Cytoscape v3.10.2. Functional enrichment analysis of the target genes was conducted using the ClusterProfiler v4.12.6 package, focusing on Gene Ontology (GO) terms and Kyoto Encyclopedia of Genes and Genomes (KEGG) pathways. Differentially expressed genes in the TCGA-PRAD cohort were identified using an adjusted *P*-value < 0.05 and an absolute log2 fold change (logFC) > 1. Univariate Cox regression analysis was performed to assess the association between differentially expressed genes and overall survival (OS) in PCa patients, with statistical significance set at *P* < .05.

### 2.4. Pan-cancer expression and prognostic analysis of miRNA

To systematically evaluate the pan-cancer dysregulation and clinical relevance of candidate miRNAs, we utilized the ENCORI Pan-Cancer Analysis Platform (https://starbase.sysu.edu.cn/), an integrated resource for RNA interactome studies that consolidates multi-omics data from 32 cancer types in TCGA and other large-scale cohorts. Differential expression analysis was performed across 17 malignancies (including BLCA, BRCA, ESCA, and PDAC), comparing tumor tissues with matched normal samples to identify consistently up/downregulated miRNAs with potential oncogenic or tumor-suppressive roles. Survival analysis employed Cox proportional hazards models to assess miRNA-prognosis associations in 32 cancer types, with stringent correction for multiple testing (FDR < 0.05).

### 2.5. Statistical and computational tools

All statistical analyses were performed using R (version 4.3.1). The TwoSampleMR package was used for MR analysis, while miRNet and Cytoscape facilitated target gene identification and network visualization, respectively. Functional enrichment and survival analyses were conducted using the ClusterProfiler and survival packages in R. Data visualization was performed using ggplot2 package.

## 3. Results

### 3.1. Five serum miRNAs are beneficial for PCa

**Figure 1.**
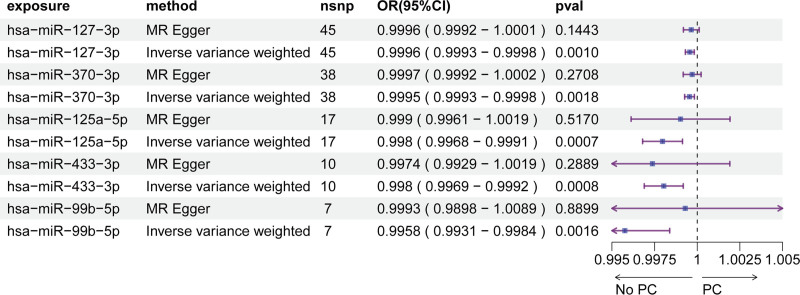
MR analysis of serum miRNAs associated with prostate cancer risk.

In the discovery cohort, serum eQTL screening identified 9500 SNPs spanning 75 miRNAs for MR analysis (Table S1, Supplementary Digital Content, https://links.lww.com/MD/P555). Using IVW MR, we found robust evidence that genetically predicted levels of 5 serum miRNAs were inversely associated with PCa risk (Fig. 1, Table S2, Supplementary Digital Content, https://links.lww.com/MD/P555): hsa-miR-127-3p: OR = 0.9996 per unit increase (95% CI: 0.9993–0.9998, *P* = .0010); hsa-miR-370-3p: OR = 0.9995 (95% CI: 0.9993–0.9998, *P* = .0018); hsa-miR-125a-p: Most significant effect (OR = 0.998, 95% CI: 0.9968–0.9991, *P* = .0007); hsa-miR-433-3p: OR = 0.998 (95% CI: 0.9969–0.9992, *P* = .0008); hsa-miR-99b-5p: Strongest magnitude of effect (OR = 0.9958, 95% CI: 0.9931–0.9984, *P* = .0016). MR-Egger regression revealed no horizontal pleiotropy (intercept *P* > .05 for all miRNAs; Table S3, Supplementary Digital Content, https://links.lww.com/MD/P555), supporting the robustness of causal estimates. Leave-one-out analysis confirmed that no individual SNP disproportionately influenced the observed associations (Fig. S1, Supplemental Digital Content, https://links.lww.com/MD/P556).

### 3.2. Identification of miRNA targets and their clinical relevance in PCa

**Figure 2.**
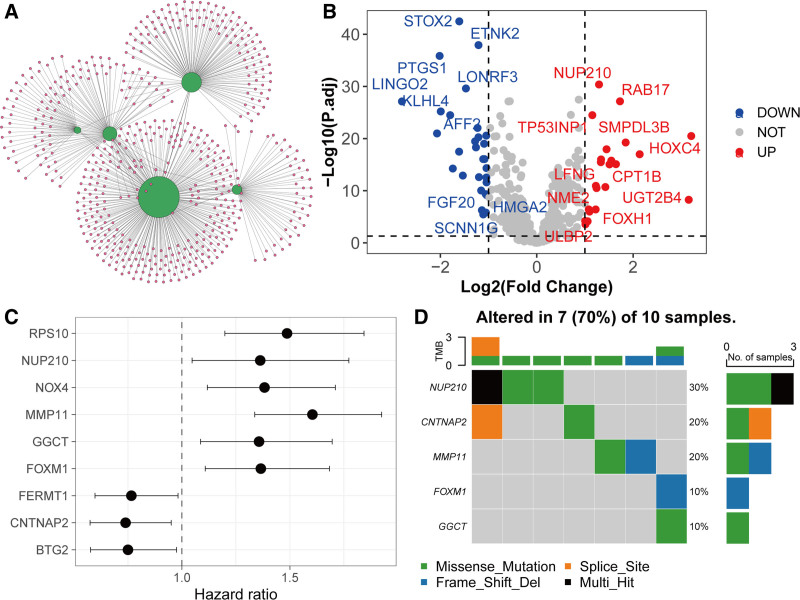
Target analysis of causal miRNAs in PCa. (A) Targets of the 5 causal miRNAs retrieved from the miRTarBase database. (B) Volcano plot of differential expression analysis of the targets of causal miRNAs in the transcriptomic data of the TCGA-PRAD cohort. (C) Univariate Cox regression analysis identified 9 targets significantly associated with OS in PCa. (D) Somatic mutation profiles of the prognostic-related targets.

**Figure 3.**
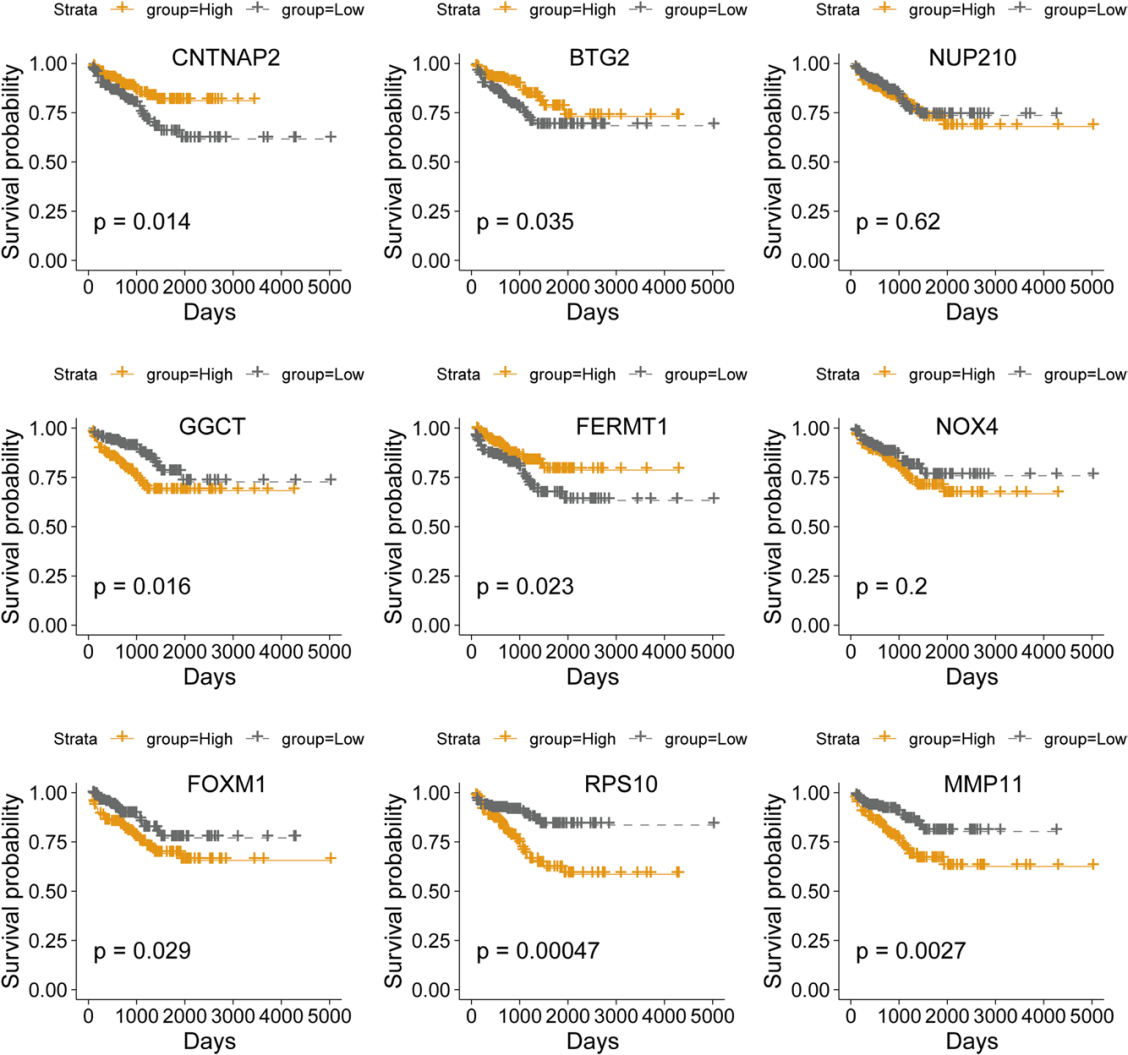
Kaplan–Meier survival curves for prognostic miRNA-target genes.

Using the miRTarBase 2025 database, which integrates experimentally validated miRNA-target interactions (MTIs) and disease-specific regulatory networks, we identified 553 target genes for 5 causal miRNAs (Fig. 2A). Notably, hsa-miR-125a-5p exhibited the broadest regulatory influence with 280 targets, consistent with prior reports of its role in oncogenic pathways. Subsequent differential expression analysis in PCa tissues revealed 22 significantly upregulated genes (e.g., MMP11, FOXM1) and 29 downregulated genes (e.g., BTG2, FERMT1) (Fig. 2B). Univariable Cox regression analysis identified 9 genes significantly associated with OS (Fig. 2C and Table S4, Supplementary Digital Content, https://links.lww.com/MD/P555), including 6 high-risk genes (RPS10, NUP210, NOX4, MMP11, GGCT, FOXM1; HR > 1) and 3 low-risk genes (FERMT1, CNTNAP2, BTG2; HR < 1). Kaplan–Meier survival curves confirmed that patients with elevated CNTNAP2, BTG2, or FERMT1 expression had significantly better OS, whereas high GGCT, FOXM1, RPS10, or MMP11 levels predicted poorer outcomes (Fig. 3). Somatic mutation profiling revealed 5 genes (NUP210, CNTNAP2, etc) with recurrent missense mutations, predominantly in domains critical for protein function (Fig. 2D). Co-expression analysis demonstrated significant negative correlations between hsa-miR-125a-5p and GGCT/MMP11 (Pearson *r* = −0.208/−0.119; *P* < .001) and between hsa-miR-99b-5p and RPS10 (r = −0.096; *P* = .0331) (Table 1). These inverse relationships suggest direct regulatory interactions, consistent with mechanistic studies showing miRNA-mediated suppression of oncogenic targets.

**Table 1:** Co-expression trends of genes associated with OS in PCa and their corresponding miRNAs.

ID	Target	Literature	Coefficient-*R*	*P*-value
hsa-miR-125a-5p	BTG2	24398324 21572407 26701625	0.01	8.27E−01
hsa-miR-125a-5p	FERMT1	23622248	0.028	5.27E**−**01
**hsa-miR-125a-5p**	**GGCT**	**23622248**	**−0.208**	**3.02E−06**
**hsa-miR-125a-5p**	**MMP11**	**22768249**	**−0.119**	**7.95E−03**
hsa-miR-370-3p	FOXM1	24055400 23576572 22900969 26617733	0.022	6.33E−01
hsa-miR-370-3p	NUP210	23313552	−0.029	5.17E−01
hsa-miR-99b-5p	CNTNAP2	23622248	−0.058	1.94E−01
hsa-miR-99b-5p	NOX4	25378491	0.003	9.55E−01
**hsa-miR-99b-5p**	**RPS10**	**23622248**	**−0.096**	**3.31E−02**

The bold font indicates that the miRNA and its target expression levels in that row are significantly negatively correlated.

OS = overall survival, PCa = prostate cancer.

### 3.3. Biological processes and pathways mediated by causal miRNAs

**Figure 4.**
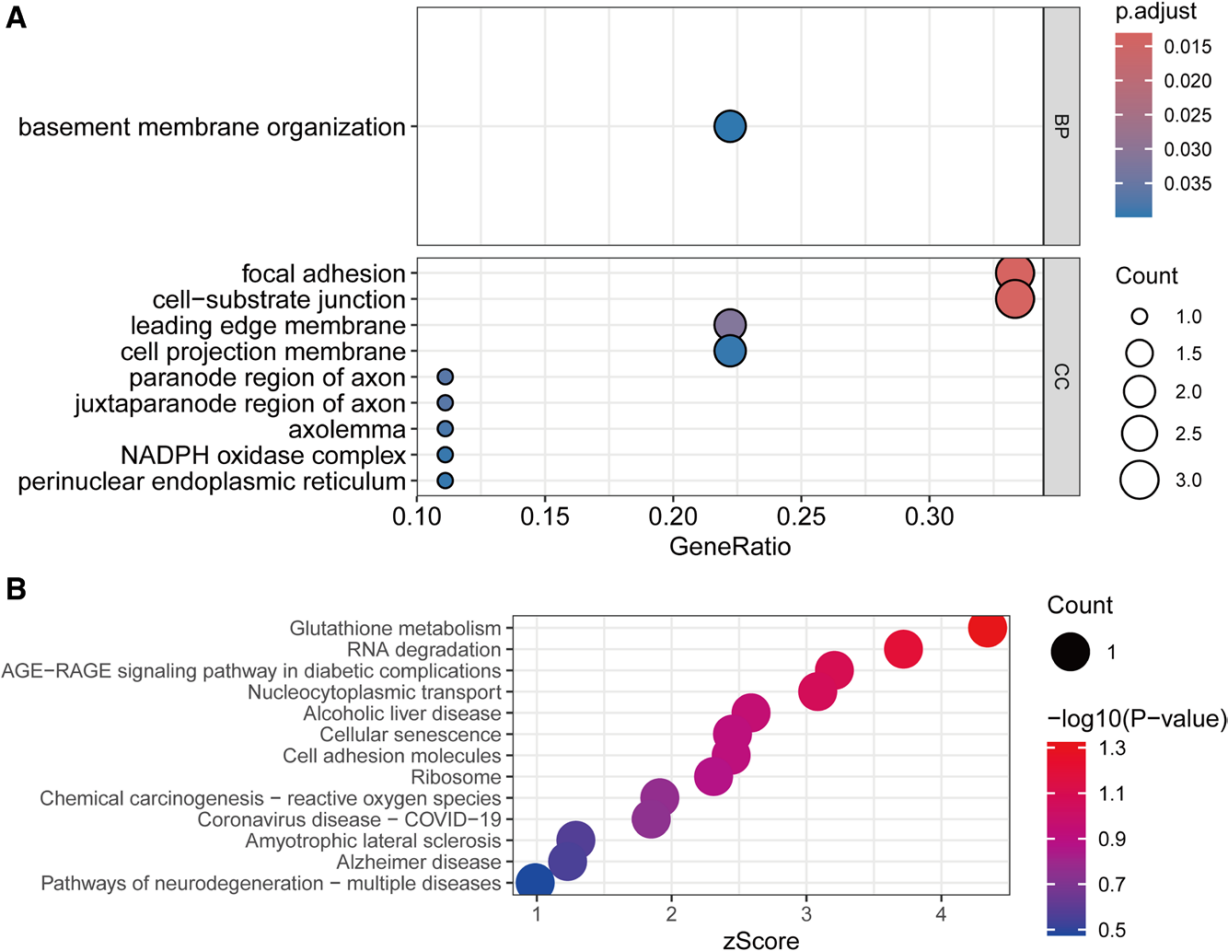
Enrichment analysis of miRNA targets associated with OS in PCa. (A) Display of GO annotation enrichment analysis. (B) Display of KEGG pathway enrichment analysis.

Enrichment analysis was conducted to elucidate the biological processes and pathways associated with the identified prognostic targets. GO analysis revealed robust enrichment in basement membrane organization. Cellular component annotation highlighted their predominant localization to focal adhesion complexes, cell-substrate junctions, and membrane protrusions (e.g., leading edge and cell projection membranes) (Fig. 4A), suggesting roles in cytoskeletal dynamics and cell motility. KEGG pathway analysis further identified significant associations with redox homeostasis (glutathione metabolism), RNA quality control (RNA degradation), senescence-associated secretory phenotypes (cellular senescence), immune modulation (cell adhesion molecules), and translational regulation (ribosome pathways) (Fig. 4B). These findings collectively underscore the multifunctional nature of the prognostic targets, spanning extracellular matrix (ECM) remodeling, stress adaptation, and therapeutic evasion mechanisms in advanced prostate malignancies.

### 3.4. Expression and prognostic correlations of causal miRNAs in cancers

Utilizing the ENCORI Pan-Cancer Analysis Platform, we systematically investigated the role of causal miRNAs in oncogenesis. Differential expression analysis across 32 cancer types (Table S5, Supplementary Digital Content, https://links.lww.com/MD/P555) identified significant dysregulation of candidate miRNAs, with hsa-miR-127-3p showing marked downregulation in PCa compared to adjacent normal tissues (fold change: 0.7, *P* < .001; Table 2). Univariate cox regression analysis revealed no statistically significant association between the 5 candidate miRNAs and OS in PRAD (*P* > .05; Table 3). Intriguingly, pan-cancer analysis demonstrated that these miRNAs independently predicted clinical outcomes in multiple cancers, such as Head and Neck Squamous Cell Carcinoma, Kidney Renal Papillary Cell Carcinoma, and Brain Lower Grade Glioma (Table S6, Supplementary Digital Content, https://links.lww.com/MD/P555).

**Table 2:** Expression analysis of causal miRNA in PCa.

miRNAs	CancerNum	NormalNum	CancerExp	NormalExp	FoldChange	*P*-value	FDR
**hsa-miR-127-3p**	**495**	**52**	**353.96**	**505.37**	**0.7**	**8.40E−08**	**1.20E-06**
hsa-miR-370-3p	495	52	2.2	3.22	0.68	.089	0.33
hsa-miR-125a-5p	495	52	546.41	515.1	1.06	.53	0.82
hsa-miR-433-3p	495	52	0.68	0.61	1.1	.51	0.82
hsa-miR-99b-5p	495	52	15,589.93	14,763.84	1.06	.63	0.82

FDR = false discovery rate, OS = overall survival.

**Table 3:** Correlation analysis of causal miRNAs with OS in PCa.

miRNAs	CancerNum	Median	coef	HR	*P*-value
hsa-miR-127-3p	495	311.72	−1.21	0.3	.11
hsa-miR-370-3p	495	2	−0.74	0.48	.28
hsa-miR-125a-5p	495	497.43	0.47	1.6	.51
hsa-miR-433-3p	495	0.52	0.66	1.94	.33
hsa-miR-99b-5p	495	14,861.23	−0.3	0.74	.64

OS = overall survival, PCa = prostate cancer.

### 3.5. Two beneficial causal miRNAs were validated in an independent cohort

**Figure 5.**
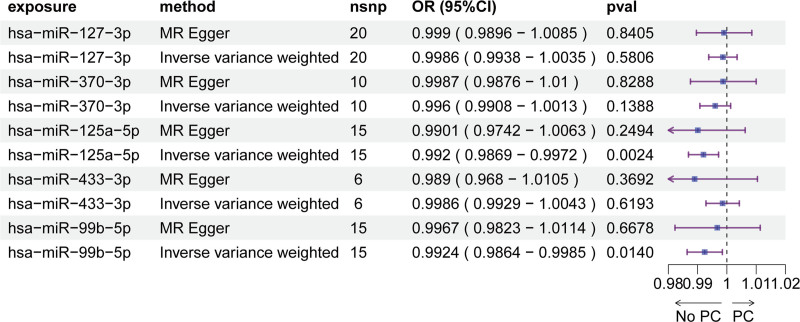
Validation of causal miRNAs in an independent cohort.

Our MR analysis leveraging the Nikpay et al cohort revealed 2 circulating microRNAs with genetically predicted causal associations with PCa risk (Fig. 5). Using IVW MR, we identified hsa-miR-125a-5p (OR = 0.992 per unit increase, 95% CI: 0.9869–0.9972, *P* = .0024) and hsa-miR-99b-5p (OR = 0.9924, 95% CI: 0.9864–0.9985, *P* = .0140) as inversely associated with PCa susceptibility. These findings align with the observation in the discovery cohort. Methodological robustness was confirmed via MR-Egger regression, which showed no evidence of horizontal pleiotropy (intercept *P* > .05 for all miRNAs, Table 4). Leave-one-out sensitivity analyses further validated that no single genetic instrument disproportionately drove the associations (Fig. S2, Supplemental Digital Content, https://links.lww.com/MD/P556). This consistency reinforces the reliability of our causal estimates.

**Table 4:** Pleiotropic effects in the validation cohort.

Exposure	egger_intercept	se	*P*-value
hsa-miR-127-3p	−9.22E−05	0.000209581	.662032653
hsa-miR-370-3p	−0.000159049	0.000229479	.492701509
hsa-miR-125a-5p	−0.000449614	0.000575704	.446964419
hsa-miR-433-3p	0.000287884	0.000968822	.773922611
hsa-miR-99b-5p	−0.000951188	0.001248101	.48038055

## 4. Discussion

This study represents the first comprehensive MR analysis to systematically investigate the causal relationships between circulating miRNAs and PCa risk. By leveraging 2 independent plasma miRNA-eQTL datasets and integrating multi-omics data from TCGA, we identified 5 miRNAs with protective effects against PCa pathogenesis, 2 of which (hsa-miR-125a-5p and hsa-miR-99b-5p) were robustly validated in an external cohort. Our findings advance current understanding of miRNA-mediated regulatory networks in PCa while providing a methodological framework for biomarker discovery using genetic instruments.

The inverse association between hsa-miR-125a-5p levels and PCa risk is consistent with its established role as a tumor suppressor across multiple malignancies. In cervical cancer, miR-125a-5p has been shown to inhibit tumor cell proliferation and invasion by targeting GALNT7 to suppress the activity of the EGFR/PI3K/AKT pathway.^[23]^ In a PCa model, overexpression of miR-125a-5p significantly inhibits cancer cell migration and induces apoptosis by upregulating NAIF1 and caspase-3.^[24]^ Notably, a clinical study by Li et al (2020)^[25]^ demonstrated that low expression of plasma exosomal miR-125a-5p is significantly associated with the occurrence of PCa (*P* = .032) and that it has a weak positive correlation with PSA levels (*R* = 0.3413), suggesting that this miRNA may serve as a novel diagnostic biomarker independent of PSA. Mechanistically, the tumor-suppressive effects of miR-125a-5p are characterized by multiple targets. In addition to the PCa-related targets identified in this study, it regulates cancer stem cell phenotypes by targeting CDK6 and CDC25A in glioblastoma^[26]^ and inhibits the EMT process by targeting MUC1 in breast cancer.^[27]^ This pleiotropic nature may explain why the GO enrichment analysis in this study shows that miR-125a-5p-related genes are involved in multiple biological processes, including cell adhesion, oxidative stress, and RNA quality control. It is worth noting that although miR-125a-5p functions as a tumor suppressor in most solid tumors, its expression levels in PCa tissues are not significantly different from those in normal tissues (Table 2), suggesting that circulating miR-125a-5p may primarily originate from secretory regulation in the tumor microenvironment or pre-metastatic niche rather than from the expression of tumor cells themselves. This hypothesis is consistent with recent studies on the spatiotemporal heterogeneity of tumor-derived exosomal miRNAs.^[11]^

In this study, miR-99b-5p was found to have protective effects against PCa at both the genetic prediction and expression validation levels, which is highly consistent with the tumor-suppressive functions revealed by multiple studies in recent years. Numerous experimental evidences indicate that miR-99b-5p plays a key role by targeting the mTOR/AR signaling axis – in PCa cells, overexpression of miR-99b-5p can simultaneously inhibit the nuclear translocation of mTOR and the androgen receptor (AR), thereby downregulating EMT markers such as Snail/N-cadherin and significantly suppressing tumor metastasis.^[28]^ Notably, this regulation exhibits racial specificity; in PCa among African Americans, the abnormal low expression of miR-99b-5p is closely related to high nuclear mTOR activity, which may partially explain the aggressive tumor phenotype in this population.^[29]^ In addition to its direct anti-tumor effects, miR-99b-5p can also enhance chemosensitivity through exosome-mediated delivery systems, producing synergistic effects when combined with enzalutamide or abiraterone and increasing the response rate of CRPC cells to treatment by 40% to 60%.^[30]^ It is worth noting that the tumor-suppressive function of miR-99b-5p is universally applicable across multiple cancer types. In colorectal cancer, it inhibits tumor proliferation and liver metastasis by targeting FGFR3,^[31]^ and in neuroblastoma studies, miR-99b-5p has been shown to enhance the sensitivity to chemotherapeutic drugs such as doxorubicin.^[32]^ These findings complement the KEGG enrichment results of this study (involving ECM remodeling and oxidative stress pathways), suggesting that miR-99b-5p may exert cross-cancer protective effects by regulating the tumor microenvironment.

The MR-derived miRNA signatures demonstrated significant enrichment in biological processes critical to metastatic progression, including basement membrane organization and glutathione metabolism. These findings are particularly relevant given the established link between redox imbalance and treatment resistance in advanced PCa.^[33]^ The identification of GGCT – a key enzyme in glutathione homeostasis – as a miR-125a-5p target provides mechanistic insights into how miRNA dysregulation may promote therapeutic evasion.^[34]^ Furthermore, the prognostic significance of co-expressed genes like MMP11 underscores the clinical relevance of these regulatory networks, as both genes have been associated with metastatic potential in hormone-sensitive PCa.^[35]^ In addition, it was reported that MMP11 may alter the stromal microenvironment of PCa to stimulate tumor angiogenesis.^[36]^

Methodologically, our 2-stage MR design addressed critical limitations of conventional observational studies by minimizing confounding and reverse causation. The consistency of effect estimates across discovery and validation cohorts (OR range: 0.992–0.9996) strengthens causal inference, though the modest effect sizes suggest these miRNAs may function synergistically within larger regulatory networks. This hypothesis is supported by the multi-target nature of miRNA regulation, as evidenced by the 553 identified target genes in our network analysis. The development of combinatorial miRNA panels, potentially integrated with PSA metrics, could enhance diagnostic specificity while addressing the “one-to-many” challenge inherent in single miRNA biomarkers.

Several limitations warrant consideration. First, the European ancestry of included cohorts limits generalizability to diverse populations with distinct genetic and environmental risk profiles. Second, while MR reduces confounding, residual pleiotropy may persist despite rigorous sensitivity analyses. Third, the low abundance of circulating miRNAs introduces technical variability in detection methods, necessitating standardization protocols for clinical translation 9. Future studies should incorporate longitudinal measurements to assess dynamic changes during disease progression and treatment response.

## 5. Conclusion

In conclusion, this MR study establishes genetically predicted levels of hsa-miR-125a-5p and hsa-miR-99b-5p as causal protective factors against PCa development. The integrated multi-omics approach elucidates their roles in regulating metastatic pathways and oxidative stress adaptation, providing a mechanistic foundation for therapeutic targeting. Validation of these miRNAs in prospective cohorts and functional characterization of prioritized targets (e.g., FOXM1-MMP11 axis) could accelerate their translation into clinical practice as noninvasive biomarkers and precision oncology tools.

## Author contributions

**Conceptualization:** Zhicheng Cong, Dandan Qiu, Haiping Hu.

**Data curation:** Zhicheng Cong, Dandan Qiu, Haiping Hu.

**Formal analysis:** Zhicheng Cong, Dandan Qiu, Haiping Hu.

**Funding acquisition:** Zhicheng Cong, Dandan Qiu, Haiping Hu.

**Investigation:** Zhicheng Cong, Dandan Qiu, Haiping Hu.

**Methodology:** Zhicheng Cong, Dandan Qiu, Haiping Hu.

**Project administration:** Zhicheng Cong, Dandan Qiu, Haiping Hu.

**Resources:** Zhicheng Cong, Dandan Qiu, Haiping Hu.

**Software:** Zhicheng Cong, Dandan Qiu, Haiping Hu.

**Supervision:** Zhicheng Cong, Dandan Qiu, Haiping Hu.

**Validation:** Zhicheng Cong, Dandan Qiu, Haiping Hu.

**Visualization:** Zhicheng Cong, Dandan Qiu, Haiping Hu.

**Writing – original draft:** Zhicheng Cong, Dandan Qiu, Haiping Hu.

**Writing – review & editing:** Zhicheng Cong, Dandan Qiu, Haiping Hu.

## Supplementary Material


