# Supplementary material for: Mendelian randomization reveals causal circulatory microRNAs in prostate cancer pathogenesis and prognosis
Source: Medicine (Baltimore). 2025 Aug 1;104(31):e43613. doi: 10.1097/MD.0000000000043613 (PMC12323976; doi:10.1097/MD.0000000000043613)
Supplement: Supplementary file 2 [file medi-104-e43613-s002.docx]

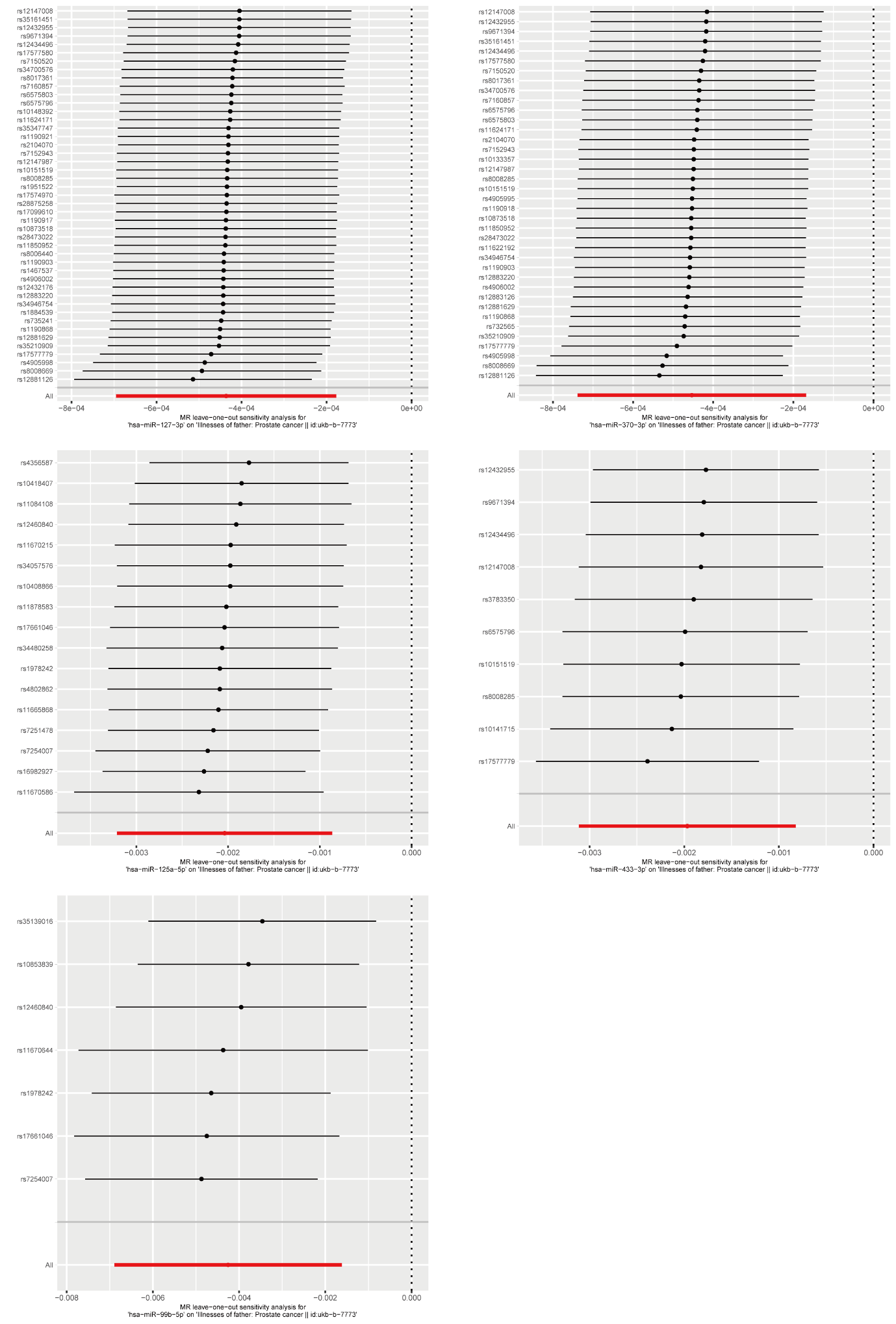


**Figure S1**. Leave-One-Out Sensitivity Analysis in the Discovery Cohort.


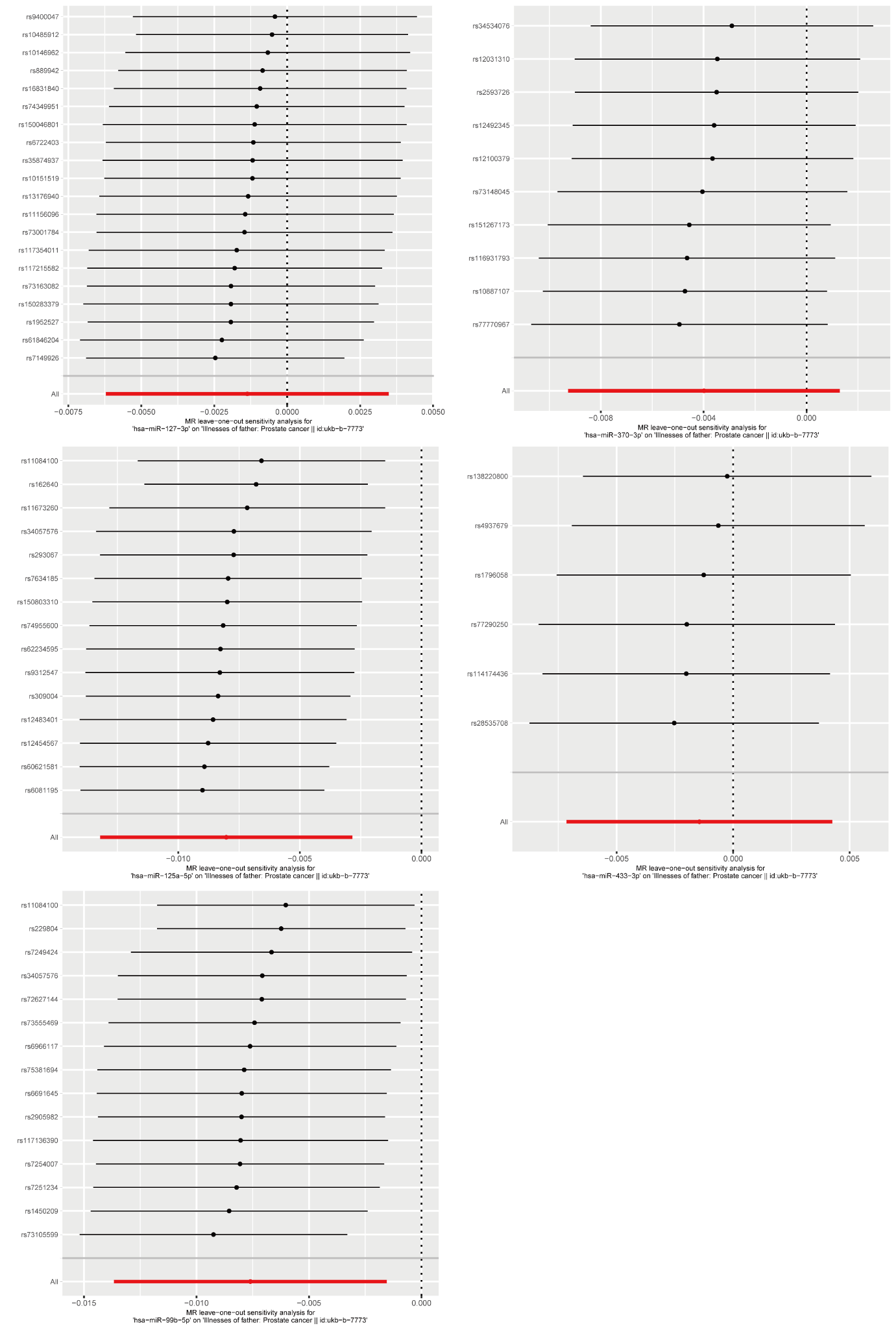


**Figure S2**. Leave-One-Out Sensitivity Analysis in the Validation Cohort.
